# Abnormal prenatal brain development in Chiari II malformation

**DOI:** 10.3389/fnana.2023.1116948

**Published:** 2023-04-17

**Authors:** Olivia Masse, Emily Kraft, Esha Ahmad, Caitlin K. Rollins, Clemente Velasco-Annis, Edward Yang, Simon Keith Warfield, Alireza A. Shamshirsaz, Ali Gholipour, Henry A. Feldman, Judy Estroff, Patricia Ellen Grant, Lana Vasung

**Affiliations:** ^1^Division of Newborn Medicine, Boston Children’s Hospital, Harvard Medical School, Boston, MA, United States; ^2^Department of Neurology Medicine, Boston Children’s Hospital, Harvard Medical School, Boston, MA, United States; ^3^Department of Radiology, Boston Children’s Hospital, Harvard Medical School, Boston, MA, United States; ^4^Maternal Fetal Care Center, Boston Children’s Hospital, Boston, MA, United States; ^5^Institutional Centers for Clinical and Translational Research, Boston Children’s Hospital, Harvard Medical School, Boston, MA, United States

**Keywords:** fetus, fetal brain, prenatal brain development, Chiari II malformation, birth defects

## Abstract

**Introduction:**

The Chiari II is a relatively common birth defect that is associated with open spinal abnormalities and is characterized by caudal migration of the posterior fossa contents through the foramen magnum. The pathophysiology of Chiari II is not entirely known, and the neurobiological substrate beyond posterior fossa findings remains unexplored. We aimed to identify brain regions altered in Chiari II fetuses between 17 and 26 GW.

**Methods:**

We used *in vivo* structural T2-weighted MRIs of 31 fetuses (6 controls and 25 cases with Chiari II).

**Results:**

The results of our study indicated altered development of diencephalon and proliferative zones (ventricular and subventricular zones) in fetuses with a Chiari II malformation compared to controls. Specifically, fetuses with Chiari II showed significantly smaller volumes of the diencephalon and significantly larger volumes of lateral ventricles and proliferative zones.

**Discussion:**

We conclude that regional brain development should be taken into consideration when evaluating prenatal brain development in fetuses with Chiari II.

## 1. Introduction

The Chiari II malformation is characterized by caudal migration of the posterior fossa contents (brain stem, cerebellum, fourth ventricle) through the foramen magnum (Cleland, 1883; Chiari, 1891, 1895). Chiari II occurs almost exclusively in fetuses with open spinal dysraphism (abnormal formation of the spine with protrusion of meninges and/or spinal nerves) most frequently found at lumbar levels (Cama et al., 1995). Chiari II is a common birth defect with an estimated incidence of approximately 0.5−1/1,000 births (Memet Özek et al., 2008; Kuhn and Emmady, 2022). Nonetheless, the exact numbers are difficult to estimate due to the increased administration of folate during pregnancy (Hidalgo et al., 2022), which may prevent this malformation, and due to unknown rates of miscarriage. For the same reasons, there are only a few prospective, longitudinal, and well-designed Chiari II studies.

In addition to the hallmark radiological findings [caudal displacement of posterior fossa content, inferior displacement of the cervical spinal cord, enlargement of ventricles, and (myelo)meningocele] in patients with Chiari II, there are a number of associated brain malformations [e.g., cerebellar hypoplasia (Van den Hof et al., 1990), collicular fusion and tectal beaking (Nagaraj et al., 2017), enlarged massa intermedia and elongation of the habenular commissure and pineal gland (Gooding et al., 1967), dysgenesis of the corpus callosum and periventricular nodular heterotopia (Hino-Shishikura et al., 2012), hypoplasia of cranial nerves and aqueductal stenosis (Tubbs and Oakes, 2013)]. In addition, Chiari II is frequently associated with secondary findings, i.e., abnormalities of the spine [e.g., platybasia (Cogan and Barrows, 1954), scoliosis (Cesmebasi et al., 2015)], spinal cord [e.g., tethered cord (Mehta et al., 2011), lipomyelomeningocele (Geerdink et al., 2012), diastematomyelia (Parmar et al., 2003)], and meninges [e.g., interdigitating gyri (Geerdink et al., 2012)]. This broad palette of associated findings supports the concept that Chiari II patients have abnormal development of the entire central nervous system (CNS) and of the non-CNS organ systems supporting it. Furthermore, important components of human fetal brain development are the transient fetal compartments, which include the ventricular zone, subventricular zone, intermediate zone, subplate zone, cortical plate, and marginal zone (Bystron et al., 2008; Kostovic and Vasung, 2009; Vasung et al., 2016). The compartments are integral for fetal development due to the events occurring within them, including cellular proliferation, migration, synaptogenesis, pruning, cell death, areal specification, and axonal myelination (Kostovic and Vasung, 2009; Kang et al., 2011). Therefore, characterizing the regional growth and development of transient fetal zones in Chiari II may be relevant to a better understanding of its pathophysiology.

Finally, even though the pathophysiology of Chiari II remains unknown, the strong association between open spinal dysraphism (i.e., lumbar meningocele and/or myelomeningocele) speaks in favor of the “CSF leak theory” (McLone and Knepper, 1989; McLone et al., 1991). According to this theory, the caudal displacement of posterior fossa contents occurs because of the cerebrospinal fluid leak at the spinal levels caused by the non-closure of the caudal end of the neuropore around 26 days of conception (Pexieder and Jelínek, 1970; McLone and Knepper, 1989). In addition, hydrocephalus and syringomyelia are two other CSF-related findings associated with Chiari II, as well as enlargement of ventricles, a prenatal finding that was recently linked with abnormal prenatal brain development (Duy et al., 2022b; Vasung et al., 2022). The size of ventricles prenatally or postnatal placement of a shunt in Chiari II patients with hydrocephalus is not linked with better neurodevelopmental outcomes (Houtrow et al., 2018). Thus, it is highly likely that something else explains worse neurodevelopmental outcomes for some cases.

Fetal MRI is currently used to quantify regional brain volumes and characterize normal (Vasung et al., 2019, 2021) and abnormal brain development (Rollins et al., 2021). Therefore, the aim of our study was to use fetal MRI to characterize the differences in spatiotemporal brain maturation between Chiari II malformation and normal fetal controls, which could serve as potential biomarkers of neurodevelopmental outcome in the future.

## 2. Materials and methods

### 2.1. Subjects

For this retrospective observational cohort study, we screened all pregnant women who had fetal MRIs acquired at Boston Children’s Hospital in the Maternal Fetal Care Center between July 2013 and July 2020. Our search query resulted in 90 cases of fetal Chiari II [including cases with additional body abnormalities]. The inclusion criteria included: Chiari II [downward herniation of cerebellum, downward displacement of the medulla, pons, and fourth ventricle, medullary kinking, abnormally shaped fourth ventricle, hypoplastic tentorium, and beaked mesencephalic tectum (Geerdink et al., 2012)]. The study was approved by the institutional review board. Due to the low prevalence and frequent association of Chiari II with brain and body abnormalities, the exclusion criteria were related to acute/subacute and chronic brain injury resulting from hypoxic/ischemic insults (e.g., placental pathology, evidence of stroke) and hemorrhage.

For the age- and sex-matched control group, we used fetal MRIs of women with healthy pregnancies who were prospectively recruited for previous studies (Gholipour et al., 2017; Rollins et al., 2021). Inclusion criteria for controls were the following: no serious maternal medical conditions (drug dependence, morbid obesity, cancer, diabetes, and gestational diabetes), fetal gestational age matching the age of cases, fetuses recruited prospectively as controls in other research studies, and fetuses with MRI acquired for reasons other than suspected malformation or injury of the central nervous system that was read by both pediatric radiologists (body) and neuroradiologists (neuro) as normal. Exclusion criteria for controls included multiple gestation pregnancies, dysmorphic features on ultrasound (US) examination, brain malformations or brain lesions identified on the US, other identified organ anomalies on the US, known chromosomal abnormalities, known congenital infections, any abnormality on the fetal MRI, and referral to MRI due to the suspicion of central nervous system injury or malformation.

Finally, we included only the subjects whose fetal MRI and outputs of the MRI processing pipeline (quality of 3D reconstruction and segmentation) passed our quality check.

### 2.2. MRI

All exams were performed on a 3T Siemens Skyra MRI, except for one exam performed on a 3T GE Signa MRI, with 18 or 30 channel body coils and included T2-weighted half-Fourier acquired single-shot turbo spin-echo (HASTE) scans along orthogonal planes. T2 HASTE parameters were the following: slice thickness 2−3 mm, in-plane resolution 1 mm, repetition time 1,400 ms, echo time 100 ms, acquisition matrix size 256 × 256 mm, and 2- or 4-section interleaved slices.

#### 2.2.1. MRI pre-processing

Slice-to-volume reconstruction was performed with SVRTK (Kuklisova-Murgasova et al., 2012) using between 3 and 10 T2 stacks to produce a super-resolution image with 0.8 mm isotropic slice thickness for each subject. A deep learning model was used to generate brain segmentations of the reconstructed images to mask and isolate the brain image (Karimi et al., 2022).^1^ Masked brain reconstructions were N4 bias field corrected, intensity normalized, and finally rigidly registered to the Computational Radiology Lab’s spatio-temporal fetal brain atlas^2^ (Gholipour et al., 2017) using FLIRT (Jenkinson et al., 2002).

#### 2.2.2. MRI segmentation

The registered reconstructions were labeled using automatic multi-atlas fusion as described in Gholipour et al. (2017). In short, for each reconstructed image registered to atlas space, reference images were selected based on gestational age from a pool of 48 atlas images of varying gestational ages, including the 18 fetal brain atlas images and 30 individual subject atlases with high-quality segmentations. Only atlases closest in gestational age to the input image were chosen as multi-atlas segmentation templates. Each chosen template was non-rigidly registered to the input image creating diffeomorphic transformations which were applied to the template segmentations. Probabilistic STAPLE was then used to produce a tissue label segmentation for the image (Akhondi-Asl and Warfield, 2013). The atlas segmentation divides the brain tissue into 28 to 29 regions (depending on gestational age), including cortical plate, white matter zones, and various subcortical structures. Due to the time limitations, two raters inspected the automatic segmentations and corrected errors manually in 14 Chiari subjects. For the purpose of this analysis, several labels were combined, resulting in 10 regions that were measured (Supplementary Figures 1, 2): Cerebral hemispheres, diencephalon (thalamus and subthalamic nucleus), ganglionic eminence, lateral ventricles, and proliferative zones, all separated by left and right sides.

### 2.3. Statistical analyses

The segmentations were used to calculate volumes for the left and right sides of the five regions for each subject. Each subject provided a set of 10 volume measurements (5 regions, left, and right). In 14 Chiari subjects, the measurements were repeated by a second rater. The data were log-transformed for analysis to eliminate skew and facilitate the expression of the findings from smaller and larger regions on a common ratio scale. We fitted the data with a repeated-measures regression model consisting of the region- and hemisphere-specific mean log volumes; a region-specific fixed effect describing the ratio between Chiari and control volumes; covariate adjustment for gestational age; an interaction term adjusting for region-specific left-right differences; and random effects to account for rater variability and correlation among the 5 regions within-subject. Sex was not included as a covariate because of missing information in 2 Chiari subjects and uncertainty about balance in the control group. The total of 450 data points provided 433 residual degrees of freedom for the region-specific comparison between Chiari II and control volumes. We applied robust regression to identify outliers and down-weight extreme values, using the bisquare weighting function with 99% efficiency. Contrasts estimated on the log scale (X ± SE) were retransformed to percentages for reporting [100% × (10X–1) ± 100% × 10X × (10SE–1)]. Results were illustrated with box plots showing raw data with median, quartile limits, and “whiskers” delimiting the range of non-outliers.

Dice’s coefficient was used to evaluate the overlap between segmentations edited by the two raters.

## 3. Results

### 3.1. Subjects

In our final analyses, we included 25 Chiari II subjects (12 females, 11 males, and 2 with no sex reported on MRI or ultrasound) and six controls (2 females and 4 males). The remaining Chiari II subjects were excluded due to poor segmentation or MRI quality. The mean age of the Chiari II subjects included in our study was 20.35 ± 1.65 GW. The mean age of control subjects was 21.38 ± 1.51 GW. There was no significant difference in age between Chiari II and the control group.

From 25 subjects with Chiari II, 8 had evidence of body abnormality [rocker-bottom feet (*N* = 1), bilateral or unilateral clubfoot (*N* = 4), levocardia (*N* = 2), cardiac abnormality with small stomach (*N* = 1)]. Furthermore, 13 subjects had dysmorphic ventricles [angular shape of frontal horns of lateral ventricles (*N* = 12), dysmorphic shape (*N* = 1)], 16 had ventriculomegaly [borderline (*N* = 2), mild ventriculomegaly (*N* = 6), moderate ventriculomegaly (*N* = 7), severe ventriculomegaly (*N* = 1), intraventricular hemorrhage (*N* = 1), Table 1], 15 had abnormalities of telencephalon [abnormalities of corpus callosum including dysgenesis, hypogenesis, thinning, and foreshortened callosum (*N* = 13), microcephaly (*N* = 1), subependymal gray matter heterotopia (*N* = 2), porencephaly (*N* = 1)], and 2 had visible abnormalities of the diencephalon (prominent massa intermedia), 13 had abnormalities of bones [flattening of frontal bones (*N* = 10) or meninges absent septum pellucidum or defects of septal leafs (*N* = 2)]. Finally, all subjects included in our study had abnormalities of the spine [open spinal dysraphism (*N* = 25)] and spinal cord [myelomeningocele (*N* = 22), myelocele (*N* = 3), and hydrosyringomyelia (*N* = 1)] most commonly at lumbar or lumbosacral level.

**TABLE 1:** Additional abnormalities (aside from posterior fossa findings) were found on fetal MRI in Chiari II fetuses.

	Body abnormality	Dysmorphic ventricles	Ventriculomegaly	Abnormalities of telencephalon	Bone or meningeal abnormalities
N (%)	8 (32%)	13 (52%)	17 (84%)	15 (60%)	13 (52%)

### 3.2. Segmentation and dice coefficients

To evaluate the overlap between structure segmentation of the two raters, Dice similarity coefficient was calculated between the two raters’ structure segmentations in all subjects and then averaged across subjects. Mean Dice was as follows: Diencephalon left = 90% and right = 91%, lateral ventricle left = 96% and right = 96%, cerebral hemisphere left = 87% and right = 87%, proliferative zone left = 79% and right = 80%, and ganglionic eminence left = 94% and right = 93%.

### 3.3. Fetuses with Chiari II have significantly smaller volumes of the diencephalon and larger volumes of lateral ventricles and proliferative zones

**FIGURE 1:**
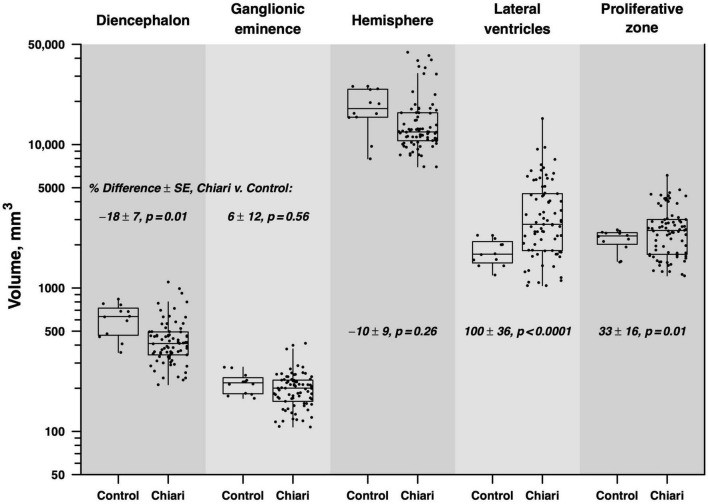
Boxes indicate the median and interquartile range (IQR), with vertical lines extending to the last data point within a distance of 1.5 × IQR from the quartile boundary. The percentage difference between mean Chiari and control volumes is indicated with standard error and test of statistical significance, derived from repeated-measures regression analysis.

Our results showed a significant difference in volumes (mm^3^) between Chiari II fetuses and the matched control group. Specifically, Chiari II subjects had significantly smaller volumes of the diencephalon (−17.8% ± 6.6), larger volumes of lateral ventricles (99.7 ± 36.3), and proliferative zones (32.5% ± 16.1). We did not find significant differences in volumes of ganglionic eminence and hemispheres between Chiari II and the control group (Figure 1). When the infants’ sex, omitted from analysis for technical reasons detailed in (the section “2. Materials and methods”), was added to the model, it showed no significant effect on volume (*p* = 0.11) or appreciable influence on the results detailed in Table 2. In the regions that showed no significant difference between Chiari II and control volume, *post hoc* power calculations based on the standard errors listed in Table 2 showed that the available sample (6 control, 25 Chiari II) provided 80% power to detect a true underlying difference as small as 25−30%.

**TABLE 2:** The volume of segmented clusters in the Chiari II and controls [cc], and comparison between two groups (% difference) with standard errors (SE), *t*-values, and *p*-values from the regression model.

	Chiari ± SE	Control ± SE	Difference (%) ± SE	*t*-value	*P*-value
**Region**					
Diencephalon	425.3 @ 14.3	517.4 @ 37.0	-17.8 @ 6.6	-2.55	0.01
Ganglionic eminence	200.3 @ 8.6	188.3 @ 18.9	6.4 @ 11.7	0.59	0.56
Hemisphere	13,770 @ 522	15,238 @ 1,292	-9.6 @ 8.5	-1.13	0.26
Lateral ventricle	3,083 @ 211	1,544 @ 255	99.7 @ 36.3	4.14	<0.01
Proliferative zones	2,501 @ 118	1,888 @ 209	32.5 @ 16.1	2.45	0.01

## 4. Discussion

In this retrospective study, we used fetal MRI and an innovative fetal MRI processing approach (semiautomatic MRI segmentation based on fetal MRI atlases) to underpin differences in fetal brain development between Chiari II and controls. Our study showed, for the first time, that aside from the known dilatation of the lateral ventricles, fetuses with Chiari II have significantly smaller volumes of the diencephalon and larger volumes of proliferative compartments (ventricular and subventricular zones).

### 4.1. Ventriculomegaly and abnormal volumes of proliferative zones in Chiari II

#### 4.1.1. There is a large variation in the severity of ventriculomegaly among 17−23 GW fetuses with Chiari II

Although dilatation of lateral ventricles is a frequent prenatal finding in Chiari II subjects, our study indicates that ventriculomegaly is not found in every fetus with Chiari II, at least between 17 and 23 GW (Figure 1). There are two potential explanations for our findings.

First, most literature reporting ventriculomegaly in Chiari II relies on estimating the size of lateral ventricles based on the atrial diameter. Since atrial diameter remains stable from 14 GW until birth [pooled average of 6.4 ± 1.2 mm (Almog et al., 2003)], it is a useful measure for a rough estimation of the severity of ventriculomegaly. According to guidelines of the International Society of Ultrasound in Obstetrics and Gynecology (International Society of Ultrasound in Obstetrics & Gynecology Education Committee, 2007; Salomon et al., 2007), an atrial diameter ≥10 mm is used to diagnose fetal ventriculomegaly, which is further classified into borderline (AD = 10 mm), mild (AD = 10−12 mm), moderate (12−15 mm), and severe (>15 mm). However, atrial measurements are susceptible to errors due to the orientation of the axial plane. Besides, given that the dysmorphic shape of lateral ventricles is a radiological hallmark of Chiari II, it remains unclear whether the atrial diameter is the best measure to estimate the severity of its ventriculomegaly.

Secondly, the atrial diameter above the critical threshold (10 mm) shows only a moderate to weak linear relationship with the volume of the lateral ventricles (Kyriakopoulou et al., 2014). Because it captures only the posterior portion of the ventricles with small differences in diameter, resulting in large differences in volume.

In our cohort, we observed a large variation in the volume of ventricles (spanning from normal to severe ventriculomegaly, Figure 1). Whether the severity of prenatal ventricular dilatation between 17 and 23 GW reflects different stages of Chiari II remains to be determined by future prospective studies that would take into account both linear and volumetric measurements of lateral ventricles.

There are numerous diverse causes of ventriculomegaly during prenatal development. Animal models of isolated ventricular enlargement offer evidence that ventricular pressures gradually increase during normal development, which is crucial for an increase in mitotic activity of neuroepithelial cells (Desmond and Jacobson, 1977; Gato and Desmond, 2009) and subsequent cortical growth and maturation. Within this framework, the CSF leak during prenatal brain development and the theoretical decrease of intraventricular pressure has been suggested to be the most important factor influencing the severity of intracranial findings in Chiari II (Righini et al., 2011). As previously mentioned, Chiari II is believed to be caused by the non-closure of the caudal end of the neuropore around 26 post-conceptual days, which results in CSF leakage (Pexieder and Jelínek, 1970; McLone and Knepper, 1989). This early egress of CSF from the cranium and spine likely leads to non-distension of the ventricles, causing displacement of posterior fossa content. However, whether the migration of posterior fossa content is the only consequence of the non-distension of ventricles remains unclear. Future longitudinal fetal MRI studies and better animal models are warranted to answer this question.

#### 4.1.2. Fetuses with Chiari II have larger proliferative compartments (ventricular and subventricular zones) than controls

Fetuses with Chiari II included in our cohort showed significantly larger volumes of proliferative compartments (ventricular and subventricular zone), likely caused by ventricular enlargement. This relationship between the increase in ventricular size and proliferative zones can be explained by considering mechanical and biological factors.

A simple increase in the diameter of a cavity (in this case, ventricles) will lead to an increase in the surface of the cavity and, consequently, to an increase in the volume of the ventricular lining (proliferative zones), i.e., if there is minimal change in thickness of proliferative zones. The current fetal imaging studies, limited by resolution, cannot show if there is an increase in the ventricular pressure that would consequently lead to a thinning of the ventricular lining (the thickness of proliferative compartments is ∼1 mm), potentially preserving its volume.

On the other hand, several recently published articles suggested the relationship between the increase in the ventricular size and a change in the structural integrity of overlying brain parenchyma might be mediated by dysregulated neural stem cell fate (Duy et al., 2022a) and epithelial cell junction pathology (Guerra et al., 2015), which could affect the regional brain volumes. The limitations of fetal MRI to discern subventricular and ventricular proliferative zones from intermediate zones are well known (Vasung et al., 2016). Thus, although we did observe a strong correlation between the volume of ventricles and proliferative zones, we cannot identify which proliferative compartments (ventricular or subventricular zone) contribute the most to this increase in volume. To this end, we cannot speculate about the biological causes behind the enlargement of proliferative zones seen in Chiari II.

Finally, an analysis of 171 children that were enrolled in the randomized control trial (Management of Myelomeningocele Study), the MOMS study, showed that there were no differences in neurodevelopmental outcomes at 30 months between children with Chiari II without postnatal hydrocephalus, children with Chiari II with non-treated postnatal hydrocephalus, and children with Chiari II with shunt-treated postnatal hydrocephalus (after taking into consideration the timing of spinal repair) (Houtrow et al., 2018). Thus, our findings of enlarged proliferative compartments in Chiari II, together with the results of the MOMS study, indicate there is reasonable doubt that fetuses in Chiari II might have abnormal brain development that could underlie higher risk for the poor neurodevelopmental outcome.

In conclusion, despite the limitations imposed by fetal MRI, we suggest that the rate of prenatal increase in ventricular volumes, which affects the size of proliferative zones before 20 GW [i.e., while the majority of neuronal precursors are still being born and are migrating toward the final destination in the cortex (Bystron et al., 2008)], should be taken into consideration when characterizing fetal brain development in Chiari II.

### 4.2. Abnormal development of diencephalon in Chiari II

Compared to controls, we found significantly smaller volumes of the diencephalon in Chiari II. Our study is the first to report this finding, suggesting the vulnerability of this region. Nonetheless, the causes leading to the altered development of diencephalon in Chiari II remain to be determined. At this point, we hypothesize that several pathophysiological mechanisms might lead to the altered development of the diencephalon.

During prenatal development, most of the diencephalon is occupied by the thalamus (ten Donkelaar and Vasung, 2015), a relay station carrying input from the periphery to the cerebral cortex. The thalamus is relevant for processing of sensory, motor, and limbic inputs. The tactile information, vibration, and position sense signals are carried by ascending sensory pathways from the spinal cord to thalamus. During embryonic development dorsal columns, receiving direct sensory information from the periphery, reach the caudal brain stem at stage 16, i.e., at about 37 postovulatory days (Müller and O’Rahilly, 1989). The second-order afferent fibers that originate from dorsal nuclei of spine ascend in the medial lemniscus to ventrobasal complex of the thalamus. In human embryos neurons of the ventrobasal complex are generated from approximately five to seven GW (Bayer, 1995). Thus, the structures relevant for transmitting tactile information, vibration, and position signals from the body to the thalamus seem to be functional very early during pregnancy. Since it is known that alteration of other signals from the periphery [such as, e.g., retinal ablation (Rakic et al., 1991)] affects the maturation of brain structures (e.g., altered cytoarchitectonic differentiation), it is possible that conditions such as Chiari II that are associated with spinal dysraphism lead to altered sensory signal transmission from the body, and consequently altered maturation of structures that serve as their relay station (notably thalamus). However, since our study only addresses the volume differences between structures, we acknowledge that the smaller volume of certain brain structures does not necessarily mean worse developmental and/or clinical outcomes. Thus, future experimental animal and prospective human studies are needed to prove these hypotheses.

### 4.3. Associated fetal abnormalities in Chiari II

The advent of fetal MR expanded the diagnostic scope of imaging in fetal Chiari II malformation (Geerdink et al., 2012) examined the reliability of 33 different morphological features associated with Chiari II malformation. The key abnormalities of Chiari II prenatally were identified as the following: downward herniation of cerebellum, downward displacement of the medulla, pons, and fourth ventricle, medullary kinking, abnormally shaped fourth ventricle, hydroplastic tentorium, and beaked mesencephalic tectum (Geerdink et al., 2012). Furthermore, the study by Geerdink demonstrated the need for further standardization with the use of morphological abnormalities detected on MR imaging guiding the diagnosis of Chiari II in a clinical and research setting. In our study, we found a large number of bone or meningeal (52%) and body abnormalities (32%) among Chiari II subjects. These additional findings lead to a question of whether certain non-CNS abnormalities might represent stigmata linked with a higher risk of poor postnatal clinical and neurodevelopmental outcomes. For example, 25% of Chiari II subjects in our study had foot abnormalities likely secondary to abnormal enervation of the lower extremities. Our recent preliminary data shows that non-syndromic isolated musculoskeletal abnormalities of the lower limbs are linked with altered structural reorganization of the brain during prenatal development (Ahmad et al., 2022). Thus, additional non-CNS abnormalities should be considered since they might be a confounder or effect modifier in studies addressing the structural brain development in Chiari II. This highlights a need for a larger prospective Chiari II study to identify an association between prenatal findings, interventions, and postnatal outcomes. Another example is the literature that postulates the association between increased Chari grade and decreased head size as a reflection of the presence of extra-axial CSF on MRI (Nagaraj et al., 2017). Such studies recognize that limited research exists comparing pre- and postnatal MRI for patients with Chiari II and emphasize the need for further investigation between MR imaging and the clinical significance of prenatal intervention.

In conclusion, there is an increasing need to continue to strengthen our understanding of the relationship between radiographic morphology and clinical severity associated with Chiari malformation so we can continue to optimize management within the pre- and postnatal periods.

### 4.4. Brain development in Chiari II and prediction of outcomes

Indications for prenatal surgery in fetuses with a Chiari II malformation are best supported by the MOMS trial. In the MOMS trial, prenatal surgical intervention was conducted prior to 26 weeks of gestation in fetuses with an open neural tube defect and outcomes were compared to outcomes of patients with routine postnatal repair. As mentioned, myelomeningocele coexists in an overwhelming majority of patients with a Chiari II malformation (Adzick et al., 2011). This clinical association is significant when considering treatment, as surgical correction of fetal myelomeningocele can decrease the severity of the Chiari II malformation in form of resolution of the hindbrain herniation and restoration of the CSF flow (Ruano et al., 2020). The trial also provided evidence supporting favorable outcomes in the prenatal-surgical group. Patients in the prenatal-surgical group were found to have statistically significant lower moderate to severe brain herniation and had morphological changes showing lower rates of brain-stem kinking, abnormal fourth-ventricle location, and syringomyelia on a 12 month clinical follow-up (Adzick et al., 2011).

Furthermore, compared to standard postnatal closure, the rates of hydrocephalus requiring CSF diversion and hindbrain herniation resolution significantly decrease with the *in utero* repair. New techniques of *in utero* closure can increase the hindbrain herniation reversal up to 90% and decrease the CSF diversion (VPS and ETV) postnatally down to only 30% of cases (Belfort et al., 2020; Flanders et al., 2020b). In addition, the treatment of hydrocephalus was significantly delayed in the fetal group compared to the postnatal group (Flanders et al., 2020a). The reversal of hindbrain herniation following fetal myelomeningocele closure subsequently reduces the incidence and severity of HH-associated brainstem dysfunction (BSD) (e.g., apnea, neurogenic dysphagia, gastroesophageal reflux disease, neuro-ophthalmologic disturbances) (Danzer et al., 2008). The long-term neurological outcome, executive functioning, and adaptive behavioral skills following fetal myelomeningocele (fMMC) surgery are significantly better than postnatal repair (Danzer et al., 2016). Also, fMMC substantially enhanced patients’ long-term neurological, cognitive, behavioral, and functional outcomes (Siahaan et al., 2023). However, currently, guidelines for surgical intervention in fetuses are based on the severity of the malformation. They do not consider additional body findings, the stage of fetal brain maturation, or changes in volume or volume trajectories of certain brain regions. Further research is necessary to deepen our understanding of the neurodevelopmental and long-term outcomes of such procedures.

## 5. Limitations

The retrospective design of our study is a limitation. The most important to mention are unknown confounders and sources of bias that could affect the selection of Chiari II cases (e.g., socioeconomic status and access to prenatal care that is associated with higher rates of prenatal MRI), quality of prenatal MRI images (suboptimal quality of the clinically acquired MRI due to the short time of MRI acquisitions and low image resolution despite fetal movements), and segmentation algorithms (optimized for *in utero* MRI segmentation of fetuses older than 20 GW). Finally, since the majority of pregnancies included in the current study ended in termination or loss to follow-up, future prospective and longitudinal imaging studies of Chiari II are warranted to better characterize brain development and disease stages in Chiari II that could be used to guide clinical decision-making and prenatal counseling. Furthermore, prenatal MRI has a limited diagnostic value in detecting certain pathologies, such as subependymal gray matter heterotopia. Thus, postnatal MRI is warranted for more precise detection of subtle brain abnormalities.

## Data availability statement

The datasets presented in this article are not readily available because of retrospectively collected patient information. Requests to access the datasets should be directed to LV, lana.vasung@childrens.harvard.edu.

## Ethics statement

The author stated that no potentially identifiable human images or data are presented in the manuscript. The study was approved by the Institutional Review Board.

## Author contributions

LV, PG, and JE: design of the study. OM, EK, EA, CR, and SW: data management and collection. OM, EK, EA, CV-A, AG, and SW: image analysis. JE, EY, and PG: MRI image interpretation. HF: statistical analysis. LV, JE, AS, and PG: interpretation of the results. All authors contributed to the preparation of the manuscript.

## Footnotes

1
https://hub.docker.com/r/davoodk/brain_extraction

2
https://doi.org/10.7910/DVN/WE9JVR

## Supplementary material

Supplementary Figure 1 Axial, coronal, and sagittal sections **(A–C)** with superimposed brain segmentations **(D–F)** in 19 GW control fetus. Color coding: hemispheres (red or brown), proliferative zones (green or blue), diencephalon (light green or purple), ventricles (light purple or sea blue), and ganglionic eminence (yellow or chartreuse).

Supplementary Figure 2 Axial, coronal, and sagittal sections **(A–C)** with superimposed brain segmentations **(D–F)** in 19 GW fetus with Chiari II. Color coding: hemispheres (red or brown), proliferative zones (green or blue), diencephalon (light green or purple), ventricles (light purple or sea blue), and ganglionic eminence (yellow or chartreuse).
