# Supplementary figures and images for: Abnormal prenatal brain development in Chiari II malformation
Source: Front Neuroanat. 2023 Apr 17;17:1116948. doi: 10.3389/fnana.2023.1116948 (PMC10149737; doi:10.3389/fnana.2023.1116948)

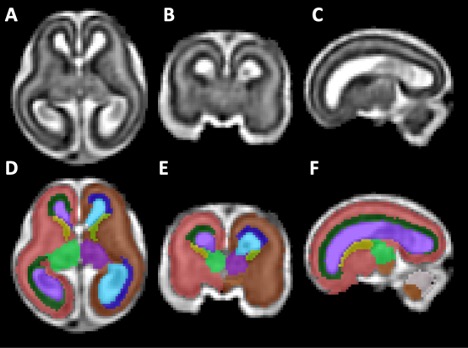

Supplement: Supplementary Figure 1 — Axial, coronal, and sagittal sections (A–C) with superimposed brain segmentations (D–F) in 19 GW control fetus. Color coding: hemispheres (red or brown), proliferative zones (green or blue), diencephalon (light green or purple), ventricles (light purple or sea blue), and ganglionic eminence (yellow or chartreuse). [file Image_1.jpg]

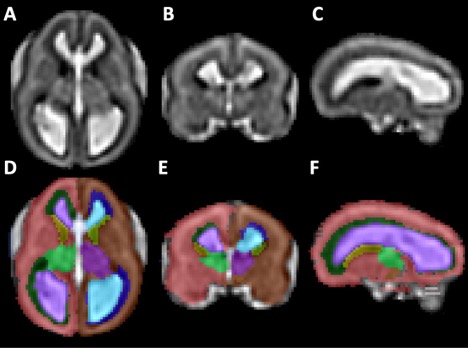

Supplement: Supplementary Figure 2 — Axial, coronal, and sagittal sections (A–C) with superimposed brain segmentations (D–F) in 19 GW fetus with Chiari II. Color coding: hemispheres (red or brown), proliferative zones (green or blue), diencephalon (light green or purple), ventricles (light purple or sea blue), and ganglionic eminence (yellow or chartreuse). [file Image_2.jpg]
